# Synthesis and Evaluation of New Oxadiazole, Thiadiazole, and Triazole Derivatives as Potential Anticancer Agents Targeting MMP-9

**DOI:** 10.3390/molecules22071109

**Published:** 2017-07-04

**Authors:** Ahmet Özdemir, Belgin Sever, Mehlika Dilek Altıntop, Halide Edip Temel, Özlem Atlı, Merve Baysal, Fatih Demirci

**Affiliations:** 1 Department of Pharmaceutical Chemistry, Faculty of Pharmacy, Anadolu University, Eskişehir 26470, Turkey; belginsever@anadolu.edu.tr (B.S.); mdaltintop@anadolu.edu.tr (M.D.A.); 2 Department of Biochemistry, Faculty of Pharmacy, Anadolu University, Eskişehir 26470, Turkey; heincedal@anadolu.edu.tr; 3 Department of Pharmaceutical Toxicology, Faculty of Pharmacy, Anadolu University, Eskişehir 26470, Turkey; oatli@anadolu.edu.tr (Ö.A.); mbaysal@anadolu.edu.tr (M.B.); 4 Department of Pharmacognosy, Faculty of Pharmacy, Anadolu University, Eskişehir 26470, Turkey; fdemirci@anadolu.edu.tr

**Keywords:** oxadiazole, thiadiazole, triazole, anticancer activity, matrix metalloproteinase, docking studies

## Abstract

Matrix metalloproteinases (MMPs) are important proteases involved in tumor progression including angiogenesis, tissue invasion, and migration. Therefore, MMPs have been reported as potential diagnostic and prognostic biomarkers in many types of cancer. New oxadiazole, thiadiazole, and triazole derivatives were synthesized and evaluated for their anticancer effects on A549 human lung adenocarcinoma and C6 rat glioma cell lines. In order to examine the relationship between their anticancer activity and MMP-9, the compounds were evaluated for their inhibitory effects on MMPs. *N*-(1,3-Benzodioxol-5-ylmethyl)-2-{[5-(((5,6,7,8-tetrahydronaphthalen-2-yl)oxy)methyl)-1,3,4-oxadiazol-2-yl]thio}acetamide (**8**) and *N*-(1,3-benzodioxol-5-ylmethyl)-2-[(5-phenyl-1,3,4-oxadiazol-2-yl)thio]acetamide (**9**) revealed promising cytotoxic effects on A549 and C6 cell lines similar to cisplatin without causing any toxicity towards NIH/3T3 mouse embryonic fibroblast cell line. Compounds **8** and **9** were also the most effective MMP-9 inhibitors in this series. Moreover, docking studies pointed out that compounds **8** and **9** had good affinity to the active site of the MMP-9 enzyme. The molecular docking and in vitro studies suggest that the MMP-9 inhibitory effects of compounds **8** and **9** may play an important role in lung adenocarcinoma and glioma treatment.

## 1. Introduction

Lung cancer is the second most commonly diagnosed cancer and the first leading cause of cancer-related deaths, accounting for about 1 in 4 cancer-related deaths in both men and women. On the other hand, brain tumors are the first leading cause of cancer death among children and adolescents [1,2,3,4].

During the last few decades, many researchers have focused on the development of anticancer drugs that target overexpression of membrane receptors and molecules involved in tumor invasion and metastasis. One of the overexpressed molecules in different malignant tumor types are matrix metalloproteinases (MMPs) [5,6,7].

MMPs, a family of zinc-dependent endoproteinases, serve pivotal functions in bone formation, vascular remodeling, and tissue allostasis. MMPs have been defined as key players in the degradation of basement membranes and extracellular matrix (ECM), which is very important for cellular behavior and widely susceptible to the proteolytic enzymes derived from cancer cells or peri-cancerous stromal cells. They have a role in normal physiologic process and also in pathological conditions such as tissue ulceration, arthritis, and cancer [7,8,9]. Correspondingly, a positive correlation has been reported between the overexpression of MMPs and poor prognosis in a variety of malignant tumors such as breast, prostate, brain, and lung cancers [10,11,12,13,14,15,16].

MMPs constitute a multigene family in humans. They are divided into six subclasses: collagenases (MMPs 1, 8 and 13), gelatinases (MMP-2 and MMP-9), stromelysins (MMPs 3, 10 and 11), matrilysins (MMPs 7 and 26), six membrane-type MMPs, and others according to their structures and substrate specificity [16]. In particular, gelatinases stimulate tumor progression by facilitating invasion, metastasis, and angiogenesis so they are prognostic factors in many solid tumors [17,18]. MMP-9 is expressed in all hematopoietic cells and fibroblasts existing in the tumor microenvironment. Besides that MMP-9 activity has been reported to increase in various tumor cells such as human lung, breast, ovarian, gastric cancers, acute myeloid leukemia, and chronic myeloid leukemia. Several lines of evidence have also pointed out that plasma and/or serum levels of MMP-9 are elevated significantly with the degree of malignancy of gliomas [19,20,21,22,23,24,25,26,27].

Apart from gelatinases, collagenases also take part in the metastasis of many tumors, but little was known about their diagnosis and prognosis. MMP-8, also known as collagenase-2 and neutrophil collagenase, is expressed by many different types of cells including neutrophils, macrophages, plasma cells, T-cells, fibroblasts, and epithelial cells. MMP-8 differs from most other MMPs with its antitumor activity due to its ability to regulate the inflammatory response caused by carcinogens. There is experimental evidence on the protective role of MMP-8 against the formation of melanoma and metastatic Lewis lung carcinoma [28,29,30,31,32,33].

1,3,4-Oxadiazoles have attracted a great deal of interest due to their unique chemical structure. These heterocycles are bioisosteres of amides and esters and they contribute to hydrogen bonding interactions with receptors. They have a broad spectrum of biological activities such as antineoplastic, antiviral, antimicrobial, anti-inflammatory, monoamine oxidase and tyrosine kinase inhibitory effects [34,35,36,37]. On the other hand, 1,3,4-thiadiazoles can easily pass cellular membranes due to their mesoionic nature and liposolubility, thus they display a variety of biological activities. To date, 1,3,4-thiadiazole based compounds have been proven to treat several cancers in vitro and in vivo by targeting the uncontrolled DNA replication/cell division, which is a hallmark of neoplastic diseases. Moreover, the heteroatoms of the thiadiazole can form interactions with biological targets including key kinases that take part in tumorigenesis [38,39,40,41].

Triazoles, containing three nitrogen atoms in a five-membered ring, show high aromatic stabilization compared to other organic compounds. They are capable of forming hydrogen bonding which is proper for many bioactive molecules. In particular, medicinal chemists focus on their anticancer activity due to their pivotal roles in anticancer drug design as nucleoside based anticancer agents, kinase inhibitors, tubulin modulators, aromatase, and sulfatase inhibitors and metal complex based antitumor agents [42,43,44].

In the light of the above findings, we aimed to synthesize new oxadiazole, thiadiazole, and triazole derivatives and evaluate their in vitro cytotoxic effects on A549 human lung adenocarcinoma and C6 rat glioma cell lines and potential inhibitory effects on gelatinases (MMP-2, MMP-9) and collagenases (MMP-1, MMP-8, MMP-13).

The modality to evaluate the selectivity of drug candidates is to test the effects of them in vitro on cultured normal fibroblasts [45]. For this purpose, the cytotoxicity of the compounds on NIH/3T3 normal mouse embryonic fibroblast cell line was investigated. Moreover, docking studies were performed to confirm the interactions of the most promising anticancer agents at the active site of the MMP-9 enzyme (PDB code: 5I12) [46].

## 2. Results and Discussion

**Scheme 1:**
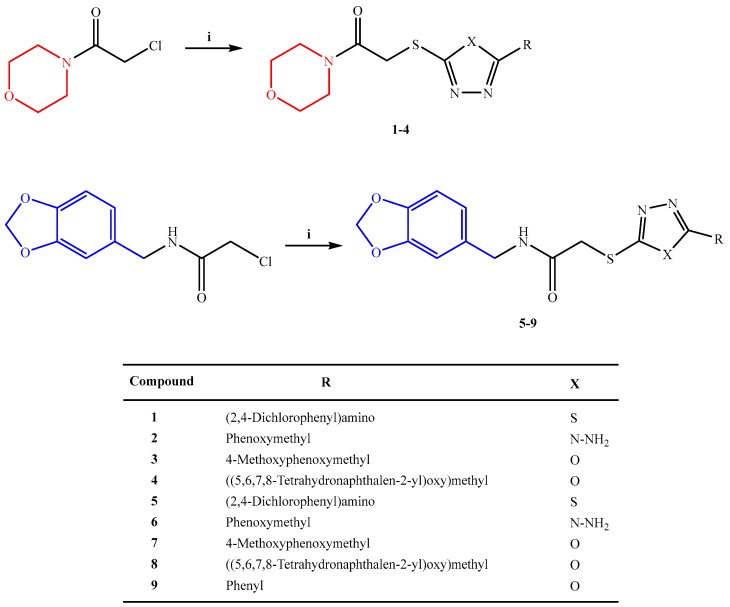
The synthetic route for the preparation of compounds **1**–**9**. Reagents and conditions: (i) Heteroaryl thiol, K_2_CO_3_, acetone, r.t., 8 h.

The synthesis of new oxadiazole, thiadiazole, and triazole derivatives (**1**–**9**) was carried out according to the steps shown in Scheme 1. Compounds **1**–**4** were obtained via the reaction of 4-(chloroacetyl)morpholine with heteroaryl thiols, whereas compounds **5**–**9** were synthesized via the reaction of *N*-(1,3-benzodioxol-5-ylmethyl)-2-chloroacetamide with heteroaryl thiols in the presence of potassium carbonate.

XTT assay was performed to investigate the antitumor effects of the compounds on A549 human lung adenocarcinoma and C6 rat glioma cell lines. Additionally, the cytotoxicity of these compounds against NIH/3T3 mouse embryonic fibroblast cell line was determined to compare their selectivity (Table 1).

Compounds **1**, **8** and **9** showed notable cytotoxic activity against C6 cell line in very similar doses compared to cisplatin (IC_50_ = 0.103 ± 0.026 mM) with IC_50_ values of 0.128 ± 0.027, 0.137 ± 0.015 and 0.157 ± 0.016 mM, respectively. Compound **3** showed anticancer activity against C6 cell line with an IC_50_ value of 0.507 ± 0.049 mM, whereas the IC_50_ values of other compounds were above the tested dose range. This result clearly indicated that heteroaryl moiety and R group had considerable influence on their antitumor effects on C6 cells. On the other hand, morpholine substituted compounds did not show any cytotoxic activity against A549 cell line, whereas only benzodioxole substituted compounds **8** and **9** showed cytotoxic effects on A549 cell line with IC_50_ values of 0.125 ± 0.020 and 0.349 ± 0.046 mM, respectively. It is important to note that (1,3-benzodioxol-5-ylmethyl)amino group enhanced antitumor activity against A549 cells. But interestingly other benzodioxole substituted compounds (**5**, **6** and **7**) did not show any cytotoxic activity against A549 cells. This outcome pointed out the importance of the oxadiazole scaffold as well as the substituent at 5th position of the oxadiazole ring for anticancer activity against A549 cells.

Selectivity index (SI) is an important indicator to determine the selectivity of the compounds [47]. In general, a selective anticancer agent can be considered as promising due to its SI value being distinctly higher than 100 [47,48]. Particularly, SI values higher than 300 indicate high selectivity against cancer cell lines [49]. According to XTT assay, compounds **1** and **9** showed low toxicity against NIH/3T3 cell line with IC_50_ values of 0.308 ± 0.037 and 0.888 ± 0.029 mM, respectively, whilst compound **8** did not cause any cytotoxic activity against NIH/3T3 cell line (IC_50_ > 1.104 mM) (Table 1). The SI values of compound **8** were found as 883.2 and 805.8, which were distinctly higher than 300, against A549 and C6 cell lines, respectively. On the other hand, the SI value of compound **9** was found as 565.6 against C6 cell line. These results pointed out the significance of compounds **8** and **9** as promising candidates to undergo further studies.

Compounds **1**, **8** and **9** were also investigated for their inhibitory effects on MMPs (MMP-1, -2, -8, -9 and -13) at 1.3 μM concentration due to their promising antiproliferative effects on A549 and C6 cell lines. Compounds **1**, **8** and **9** showed inhibitory effects on MMP-9 (24.78 ± 0.86%, 25.75 ± 2.16%, 30.46 ± 3.27%, respectively). Although compound **1** exhibited inhibitory effect on MMP-8 (15 ± 1.4%), compounds **8** and **9** showed no inhibitory activity against MMP-8. None of the compounds displayed inhibitory effects on MMP-1, MMP-2, and MMP-13. The inhibition percentages of the test compounds and *N*-isobutyl-*N*-(4-methoxyphenylsulfonyl)glycyl hydroxamic acid (NNGH) are mentioned in Table 2.

MMP-9 is produced and secreted by different types of malignant cells and inflammatory cells. Overexpression of this gelatinase implicated with tumor progression and metastasis. Inhibition of MMP-9 expression showed anti-metastatic effect in various investigations conducted on pulmonary metastasis of prostate cancer, fibrosarcomas, and melanomas [50]. One of the important mechanism associated with anticancer activity seems to be the inhibitory effect on MMP. Thus, the anticancer effects of various compounds related to MMPs have been investigated. Poudel et al. demonstrated that triticumoside suppressed migration via inhibition of MMP-2/9 in A549 cells and it was identified as a potential anti-lung cancer drug [51]. Lee et al. reported that dehydrocordaline, an alkaloid isolated from *Corydalis tutschaninovii* tuber, exerted anti-metastatic potential via the inhibition of mRNA and protein levels of MMP-7 and MMP-9 in H1299 cells [52]. Data obtained from our current work suggested that potent inhibitory effects of compounds **8** and **9** on MMP-9 enzyme may be responsible for their anticancer effects.

MMP-8, identified as an anti-target enzyme, displays antitumor activity [53]. Cancer incidence has been increased in MMP-8 knockout mice. Some studies have revealed the potential mechanisms of MMP-8 tumor suppressing functions. In one of these studies, expression of MMP-8 in tumor cells tightens their adhesion to the ECM and thereby may directly suppress metastatic behavior. Moreover, various studies suggested that tumors occur more frequently in MMP-8 deficient mice compared with wild-type control and increased inflammation delays wound healing in MMP-8 deficient mice [54,55,56].

**Figure 1:**
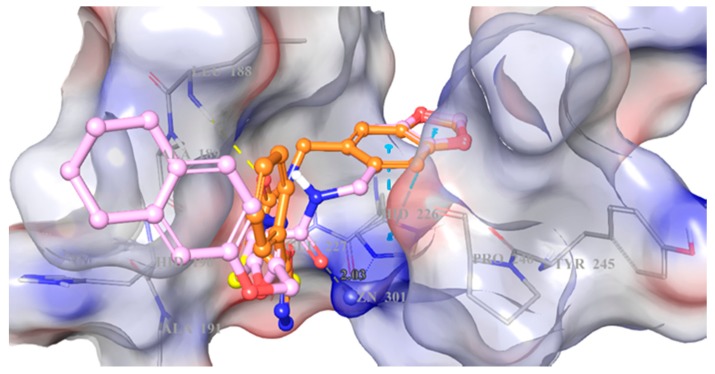
Docking positions of compounds **8** and **9** at the active site of MMP-9 (PDB code: 5I12) together (Ligand custom carbons are colored in pink for compound **8** and in orange for compound **9**; the zinc atom is displayed in grey sphere).

**Figure 2:**
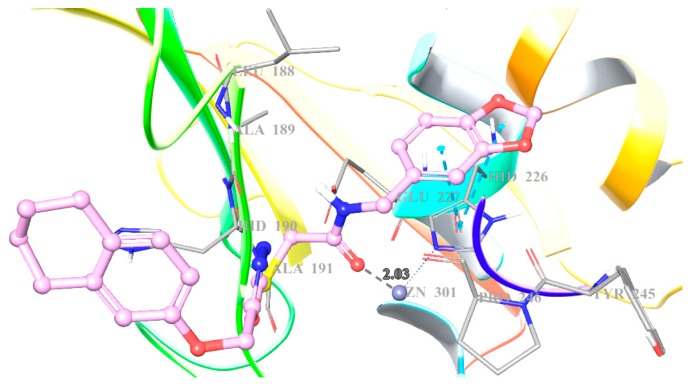

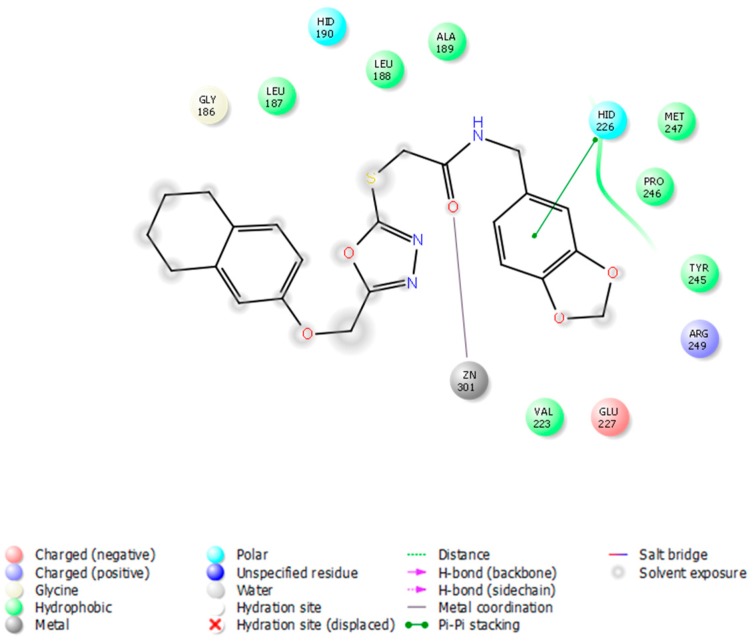
Docking position and interactions of compound **8** at the active site of MMP-9 (PDB code: 5I12), respectively. (Ligand custom carbons are colored in pink for compound **8** and the zinc atom is displayed in grey sphere).

**Figure 3:**
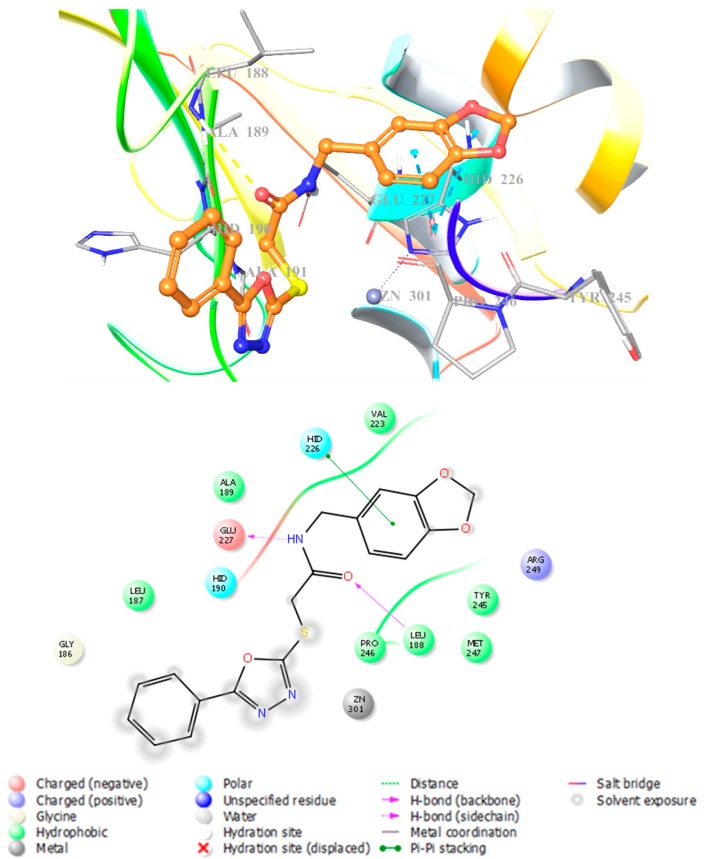
Docking position and interactions of compound **9** at the active site of MMP-9 (PDB code: 5I12), respectively. (Ligand custom carbons are colored in orange for compound **9** and the zinc atom is displayed in grey sphere).

Docking studies were also performed for compounds **8** and **9** at the active site of MMP-9 (PDB code: 5I12). Compounds **8** and **9** showed good affinity close to the active site of the enzyme that was previously defined [46]. In Figure 1, Figure 2 and Figure 3, the best docking positions of compounds **8** and **9** at the active site of MMP-9 and all interactions between these compounds and MMP-9 were shown and described. The positions of compounds **8** and **9** docked to MMP-9 were detected as similar to each other. At the MMP-9 surface, 1,3-benzodioxol-5-ylmethyl group in both compounds has pi-pi stacking contacts with His226 residue. The acetamido moiety of compound **8** is engaged in metal coordination with the Zn301, whereas the same group of compound **9** presents two H-bonds with the amino acids Glu227 and Leu188, respectively [57].

From data obtained from our study, it can be concluded that the selective inhibition of MMP-9 might play a role in anticancer effects of compounds **8** and **9**.

## 3. Materials and Methods

### 3.1. Chemistry

5-Phenyl-1,3,4-oxadiazole-2-thiol (98%) was purchased from Alfa Aesar (Karlsruhe, Germany), whereas other reagents were purchased from other commercial suppliers. Melting points (M.p.) were determined on an Electrothermal 9100 melting point apparatus (Weiss-Gallenkamp, Loughborough, UK) and are uncorrected. IR spectra were recorded on an IRPrestige-21 Fourier Transform Infrared spectrophotometer (Shimadzu, Tokyo, Japan). ^1^H-NMR and ^13^C-NMR spectra were recorded on a Varian Mercury-400 FT-NMR spectrometer (Agilent, Palo Alto, CA, USA). Mass spectra were recorded on a Shimadzu LCMS-IT-TOF system (Shimadzu, Kyoto, Japan). Thin Layer Chromatography (TLC) was performed on TLC Silica gel 60 F254 aluminium sheets (Merck, Darmstadt, Germany) to check the purity of the compounds.

#### 3.1.1. General Procedure for the Preparation of 5-[(2,4-Dichlorophenyl)amino]-1,3,4-thiadiazole-2(3*H*)-thione

The synthesis of 5-[(2,4-dichlorophenyl)amino]-1,3,4-thiadiazole-2(3*H*)-thione was carried out as described previously [58].

#### 3.1.2. General Procedure for the Preparation of 4-Amino-5-(phenoxymethyl)-2,4-dihydro-3*H*-1,2,4-triazole-3-thione

The synthesis of 4-amino-5-(phenoxymethyl)-2,4-dihydro-3*H*-1,2,4-triazole-3-thione was carried out as described previously [59].

#### 3.1.3. General Procedure for the Preparation of 5-[(4-Methoxyphenoxy)methyl]-1,3,4-oxadiazole-2(3*H*)-thione and 5-{[(5,6,7,8-tetrahydronaphthalen-2-yl)oxy]methyl}-1,3,4-oxadiazole-2(3*H*)-thione

The synthesis of 5-[(4-methoxyphenoxy)methyl]-1,3,4-oxadiazole-2(3*H*)-thione and 5-{[(5,6,7,8-tetrahydronaphthalen-2-yl)oxy]methyl}-1,3,4-oxadiazole-2(3*H*)-thione was carried out as described previously [60].

#### 3.1.4. General Procedure for the Preparation of Compounds **1**–**4**

A mixture of 4-(chloroacetyl)morpholine (2 mmol) and appropriate heteroaryl thiol (2 mmol) in acetone (10 mL) was stirred at room temperature for 8 h in the presence of potassium carbonate (2 mmol) and filtered. The residue was washed with water and crystallized from ethanol.

*2-{[5-((2,4-Dichlorophenyl)amino)-1,3,4-thiadiazol-2-yl]thio}-1-morpholinoethan-1-one* (**1**). White solid (78%). M.p.: 154 °C. IR ν_max_ (cm^−1^): 3221.12, 3192.19 (N–H stretching), 3143.97, 3091.89, 3066.82, 3034.03 (Aromatic C–H stretching), 2970.38, 2914.44, 2866.22 (Aliphatic C–H stretching), 1629.85 (C=O stretching), 1581.63, 1529.55, 1485.19, 1467.83 (N–H bending, C=N and C=C stretching), 1390.68, 1359.82 (C–H bending), 1303.88, 1271.09, 1255.66, 1220.94, 1139.93, 1107.14, 1045.42 (C–N, C–O stretching and aromatic C–H in plane bending), 964.41, 881.47, 860.25, 842.89, 821.68, 810.10, 765.74, 686.66, 653.87 (aromatic C–H out of plane bending and C–S stretching). ^1^H-NMR (400 MHz, DMSO-*d*_6_) δ (ppm): 3.43–3.49 (m, 4H, morpholine H_3_ and H_5_), 3.53–3.60 (m, 4H, morpholine H_2_ and H_6_), 4.29 (s, 2H, S-CH_2_), 7.41 and 7.43 (two d, *J* = 2.0 Hz, *J* = 2.4 Hz, 1H, aromatic), 7.62 (d, *J* = 2.4 Hz, 1H, aromatic), 8.31 (d, *J* = 9.2 Hz, 1H, aromatic), 9.92 (s, 1H, N-H). ^13^C-NMR (75 MHz, DMSO-*d*_6_): 37.49 (CH_2_), 42.50 (CH_2_), 46.35 (CH_2_), 66.42 (2CH_2_), 122.56 (CH), 123.52 (C), 127.04 (C), 128.35 (CH), 129.42 (CH), 136.59 (C), 155.13 (C), 164.1 (C), 168.2 (C). HRMS (ESI) (*m*/*z*): [M + H]^+^ calcd. for C_14_H_14_Cl_2_N_4_O_2_S_2_: 405.0008, found: 405.0002.

*2-{[4-Amino-5-(phenoxymethyl)-4H-1,2,4-triazol-3-yl]thio}-1-morpholinoethan-1-one* (**2**). White solid (67%). M.p.: 213 °C. IR ν_max_ (cm^−1^): 3325.28 (N–H stretching), 3170.97, 3064.89 (Aromatic C–H stretching), 2968.45, 2924.09, 2866.22 (Aliphatic C–H stretching), 1643.35 (C=O stretching), 1597.06, 1583.56, 1487.12, 1467.83 (N–H bending, C=N and C=C stretching), 1436.97, 1413.82, 1377.17 (C–H bending), 1280.73, 1280.73, 1242.16, 1222.87, 1190.08, 1112.93, 1043.49, 1028.06, 1012.63 (C–N, C–O stretching and aromatic C–H in plane bending), 970.19, 943.19, 910.40, 856.39, 761.88, 690.52 (aromatic C–H out of plane bending and C–S stretching). ^1^H-NMR (400 MHz, DMSO-*d*_6_) δ (ppm): 3.44 (d, *J* = 4.8 Hz, 2H, morpholine), 3.49 (d, *J* = 4.0 Hz, 2H, morpholine), 3.53 (d, *J* = 4.4 Hz, 2H, morpholine), 3.58 (d, *J* = 4.8 Hz, 2H, morpholine), 4.23 (s, 2H, S-CH_2_), 5.16 (s, 2H, Ar-OCH_2_), 6.07 (s, 2H, N-NH_2_), 6.96 (t, *J* = 7.2 Hz, *J* = 7.6 Hz, *J* = 14.8 Hz, 1H, aromatic), 7.06 (d, *J* = 8.0 Hz, 2H, aromatic), 7.30 (t, *J* = 8 Hz, *J* = 16 Hz, 2H, aromatic). ^13^C-NMR (75 MHz, DMSO-*d*_6_): 35.69 (CH_2_), 42.45 (CH_2_), 46.30 (CH_2_), 59.37 (CH_2_), 66.39 (2CH_2_), 115.34 (2CH), 121.70 (CH), 129.94 (2CH), 152.65 (C), 158.35 (2C), 166.27 (C). HRMS (ESI) (*m*/*z*): [M + H]^+^ calcd. for C_15_H_19_N_5_O_3_S: 350.1281, found: 350.1274.

*2-{[5-((4-Methoxyphenoxy)methyl)-1,3,4-oxadiazol-2-yl]thio}-1-morpholinoethan-1-one* (**3**). White solid (75%). M.p.: 119 °C. IR ν_max_ (cm^−1^): 2968.45, 2914.44, 2862.36 (Aliphatic C–H stretching), 1647.21 (C=O stretching), 1591.27, 1558.48, 1508.33, 1469.76, 1456.26 (C=N and C=C stretching), 1438.90, 1398.39, 1382.96 (C–H bending), 1284.59, 1267.23, 1217.08, 1163.08, 1111.0, 1053.13, 1024.20 (C–N, C–O stretching and aromatic C–H in plane bending), 964.41, 912.33, 854.47, 823.60, 794.67, 729.09, 696.30, 675.09, 646.15 (aromatic C–H out of plane bending and C–S stretching). ^1^H-NMR (400 MHz, DMSO-*d*_6_) δ (ppm): 3.43–3.48 (m, 4H, morpholine H_3_ and H_5_), 3.52–3.59 (m, 4H, morpholine H_2_ and H_6_), 3.69 (s, 3H, OCH_3_), 4.48 (s, 2H, S-CH_2_), 5.28 (s, 2H, Ar-OCH_2_), 6.86 (d, *J* = 9.2 Hz, 2H, aromatic), 6.98 (d, *J* = 8.8 Hz, 2H, aromatic). ^13^C-NMR (75 MHz, DMSO-*d*_6_): 36.94 (CH_2_), 42.54 (CH_2_), 46.24 (CH_2_), 55.85 (CH_3_), 60.57 (CH_2_), 66.33 (2CH_2_), 115.14 (2CH), 116.61 (2CH), 151.64 (C), 154.73 (C), 164.14 (C), 165.06 (C), 165.16 (C). HRMS (ESI) (*m*/*z*): [M + H]^+^ calcd. for C_16_H_19_N_3_O_5_S: 366.1118, found: 366.1110.

*2-{[5-(((5,6,7,8-Tetrahydronaphthalen-2-yl)oxy)methyl)-1,3,4-oxadiazol-2-yl]thio}-1-morpholinoethan-1-one* (**4**). White solid (80%). M.p.: 135 °C. IR ν_max_ (cm^−1^): 2976.16, 2929.87, 2854.65 (Aliphatic C–H stretching), 1649.14 (C=O stretching), 1633.71, 1598.99, 1498.69, 1463.97 (C=N and C=C stretching), 1446.61, 1400.32, 1381.03 (C–H bending), 1230.58, 1157.29, 1112.93, 1037.70, 1024.20 (C–N, C–O stretching and aromatic C–H in plane bending), 966.34, 921.97, 910.40, 823.60, 800.46, 694.37 (aromatic C–H out of plane bending and C–S stretching). ^1^H-NMR (400 MHz, DMSO-*d*_6_) δ (ppm): 1.66–1.69 (m, 4H, tetrahydronaphthalene H_6_ and H_7_), 2.61–2.66 (m, 4H, tetrahydronaphthalene H_5_ and H_8_), 3.42–3.49 (m, 4H, morpholine H_3_ and H_5_), 3.53–3.61 (m, 4H, morpholine H_2_ and H_6_), 4.48 (s, 2H, S-CH_2_), 5.28 (s, 2H, Ar-OCH_2_), 6.72–6.76 (m, 2H, aromatic), 6.95 (d, *J* = 8.4 Hz, 1H, aromatic). ^13^C-NMR (75 MHz, DMSO-*d*_6_): 23.05 (CH_2_), 23.31 (CH_2_), 28.42 (CH_2_), 29.48 (CH_2_), 36.93 (CH_2_), 42.54 (CH_2_), 46.24 (CH_2_), 59.84 (CH_2_), 66.33 (2CH_2_), 113.05 (CH), 115.17 (CH), 130.32 (CH), 130.43 (C), 138.33 (C), 155.46 (C), 164.13 (C), 165.05 (C), 165.16 (C). HRMS (ESI) (*m*/*z*): [M + H]^+^ calcd. for C_19_H_23_N_3_O_4_S: 390.1482, found: 390.1472.

#### 3.1.5. General Procedure for the Preparation of Compounds **5**–**9**

A mixture of *N*-(1,3-benzodioxol-5-ylmethyl)-2-chloroacetamide (2 mmol) and appropriate heteroaryl thiol (2 mmol) in acetone (10 mL) was stirred at room temperature for 8 h in the presence of potassium carbonate (2 mmol) and filtered. The residue was washed with water and crystallized from ethanol.

*N-(1,3-Benzodioxol-5-ylmethyl)-2-{[5-((2,4-dichlorophenyl)amino)-1,3,4-thiadiazol-2-yl]thio}acetamide* (**5**). White solid (76%). M.p.: 187 °C. IR ν_max_ (cm^−1^): 3253.91 (N–H stretching), 3093.82, 3034.03 (Aromatic C–H stretching), 2981.95, 2893.22 (Aliphatic C–H stretching), 1645.28 (C=O stretching), 1593.20, 1529.55, 1504.48, 1485.19, 1463.97 (N–H bending, C=N and C=C stretching), 1438.90, 1411.89, 1388.75 (C–H bending), 1301.95, 1240.23, 1099.43, 1037.70 (C–N, C–O stretching and aromatic C–H in plane bending), 931.62, 918.12, 858.32, 804.32, 767.67, 651.94 (aromatic C–H out of plane bending and C–S stretching). ^1^H-NMR (400 MHz, DMSO-*d*_6_) δ (ppm): 3.93 (s, 2H, S-CH_2_), 4.19 (d, 2H, Ar-CH_2_), 5.93 (s, 2H, O-CH_2_-O), 6.70 (d, *J* = 8.4 Hz, 1H), 6.79 (d, *J* = 7.2 Hz, 2H, aromatic), 7.42 (dd, *J* = 2.4 Hz, *J* = 6.8 Hz, 1H, aromatic), 7.62 (d, *J* = 2.0 Hz, 1H, aromatic), 8.34 (d, *J* = 8.0 Hz, 1H, aromatic), 8.62 (t, *J* = 5.2 Hz, *J* = 5.6 Hz, *J* = 10.8 Hz, 1H, CONH), 9.93 (brs, 1H, thiadiazole-NH). ^13^C-NMR (75 MHz, DMSO-*d*_6_): 37.68 (CH_2_), 42.79 (CH_2_), 101.26 (CH_2_), 108.36 (CH), 108.41 (CH), 120.89 (CH), 122.52 (CH), 123.46 (C), 126.95 (C), 128.31 (CH), 129.41 (C), 133.26 (CH), 136.64 (C), 146.55 (C), 147.71 (C), 152.70 (C), 165.41 (C), 167.12 (C). HRMS (ESI) (*m*/*z*): [M + H]^+^ calcd. for C_18_H_14_Cl_2_N_4_O_3_S_2_: 468.9957, found: 468.9956.

*2-{[4-Amino-5-(phenoxymethyl)-4H-1,2,4-triazol-3-yl]thio}-N-(1,3-benzodioxol-5-ylmethyl)acetamide* (**6**). White solid (74%). M.p.: 148 °C. IR ν_max_ (cm^−1^): 3315.63, 3234.62 (N–H stretching), 3053.32 (Aromatic C–H stretching), 2958.80, 2908.65 (Aliphatic C–H stretching), 1649.14 (C=O stretching), 1629.85, 1598.99, 1564.27, 1492.90 (N–H bending, C=N and C=C stretching), 1446.61, 1409.96, 1371.39 (C–H bending), 1325.10, 1246.02, 1219.01, 1172.72, 1099.43, 1041.56, 1010.70 (C–N, C–O stretching and aromatic C–H in plane bending), 983.70, 925.83, 873.75, 858.32, 812.03, 775.38, 748.38, 692.44, 646.15 (aromatic C–H out of plane bending and C–S stretching). ^1^H-NMR (400 MHz, DMSO-*d*_6_) δ (ppm): 3.93 (s, 2H, S-CH_2_), 4.18 (d, 2H, Ar-CH_2_), 5.15 (s, 2H, Ar-OCH_2_), 5.96 (s, 2H, O-CH_2_-O), 6.05 (s, 2H, N-NH_2_), 6.70 (d, *J* = 8.0 Hz, 1H, aromatic), 6.81 (d, *J* = 7.6 Hz, 2H, aromatic), 6.96 (t, *J* = 7.2 Hz, *J* = 14.4 Hz, 1H, aromatic), 7.06 (d, *J* = 8.0 Hz, 2H, aromatic), 7.30 (t, *J* = 7.6 Hz, *J* = 15.2 Hz, 2H, aromatic), 8.64 (brs, 1H, CONH). ^13^C-NMR (75 MHz, DMSO-*d*_6_): 35.66 (CH_2_), 42.79 (CH_2_), 59.35 (CH_2_), 101.27 (CH_2_), 108.34 (CH), 108.45 (CH), 115.33 (2CH), 120.87 (CH), 121.70 (CH), 129.94 (2CH), 133.28 (C), 146.55 (C), 147.71 (C), 152.70 (C), 152.91 (C), 158.37 (C), 167.52 (C). HRMS (ESI) (*m*/*z*): [M + H]^+^ calcd. for C_19_H_19_N_5_O_4_S: 414.1231, found: 414.1221.

*N-(1,3-Benzodioxol-5-ylmethyl)-2-{[5-((4-methoxyphenoxy)methyl)-1,3,4-oxadiazol-2-yl]thio}acetamide* (**7**). Pale yellow solid (79%). M.p.: 127 °C. IR ν_max_ (cm^−1^): 3257.77 (N–H stretching), 3074.53 (Aromatic C–H stretching), 2995.45, 2900.94, 2833.43 (Aliphatic C–H stretching), 1666.50 (C=O stretching), 1600.92, 1554.63, 1504.48, 1489.05 (N–H bending, C=N and C=C stretching), 1444.68, 1425.40, 1409.96 (C–H bending), 1321.24, 1230.58, 1159.22, 1103.28, 1072.42, 1035.77 (C–N, C–O stretching and aromatic C–H in plane bending), 921.97, 852.54, 815.89, 781.17, 725.23, 684.73, 653.87 (aromatic C–H out of plane bending and C–S stretching). ^1^H-NMR (400 MHz, DMSO-*d*_6_) δ (ppm): 3.68 (s, 3H, OCH_3_), 4.10 (s, 2H, S-CH_2_), 4.19 (d, *J* = 6.0 Hz, 2H, Ar-CH_2_), 5.27 (s, 2H, Ar-OCH_2_), 5.95 (s, 2H, O-CH_2_-O), 6.72 (d, *J* = 8.0 Hz, 1H, aromatic), 6.80–6.88 (m, 4H, aromatic), 6.97 (dt, *J* = 2.4 Hz, *J* = 4.0 Hz, *J* = 8.8 Hz, 2H, aromatic), 8.71 (t, *J* = 5.2 Hz, *J* = 6.0 Hz, *J* = 11.2 Hz, 1H, CONH). ^13^C-NMR (75 MHz, DMSO-*d*_6_): 36.15 (CH_2_), 42.89 (CH_2_), 55.85 (CH_3_), 60.57 (CH_2_), 101.29 (CH_2_), 108.38 (CH), 108.46 (CH), 115.14 (2CH), 116.59 (2CH), 120.94 (CH), 133.14 (C), 146.59 (C), 147.72 (C), 151.65 (C), 154.73 (C), 164.23 (C), 164.94 (C), 166.21 (C). HRMS (ESI) (*m*/*z*): [M + H]^+^ calcd. for C_20_H_19_N_3_O_6_S: 430.1067, found: 430.1064.

*N-(1,3-Benzodioxol-5-ylmethyl)-2-{[5-(((5,6,7,8-tetrahydronaphthalen-2-yl)oxy)methyl)-1,3,4-oxadiazol-2-yl]thio}acetamide* (**8**). Ivory solid (73%). M.p.: 96 °C. IR ν_max_ (cm^−1^): 3292.49 (N–H stretching), 2926.01, 2856.58 (Aliphatic C–H stretching), 1681.93 (C=O stretching), 1664.57, 1529.55, 1500.62, 1485.19 (N–H bending, C=N and C=C stretching), 1442.75, 1386.82, 1369.46 (C–H bending), 1325.10, 1247.94, 1236.37, 1163.08, 1122.57, 1099.43, 1037.70 (C–N, C–O stretching and aromatic C–H in plane bending), 925.83, 862.18, 802.39, 771.53, 686.66, 665.44 (aromatic C–H out of plane bending and C–S stretching). ^1^H-NMR (400 MHz, DMSO-*d*_6_) δ (ppm): 1.67 (s, 4H, tetrahydronaphthalene H_6_ and H_7_), 2.61–2.65 (m, 4H, tetrahydronaphthalene H_5_ and H_8_), 4.10 (s, 2H, S-CH_2_), 4.18 (s, 1H, Ar-CH), 4.53 (s, 1H, Ar-CH), 4.72 (s, 1H, Ar-O-CH), 5.27 (s, 1H, Ar-O-CH), 5.95 (s, 2H, O-CH_2_-O), 6.72-6.94 (m, 6H, aromatic), 8.78 (brs, 1H, CONH). ^13^C-NMR (75 MHz, DMSO-*d*_6_): 23.04 (CH_2_), 23.31 (CH_2_), 28.43 (CH_2_), 29.47 (CH_2_), 36.15 (CH_2_), 42.87 (CH_2_), 59.85 (CH_2_), 101.28 (CH_2_), 108.38 (CH), 108.44 (CH), 113.04 (CH), 115.16 (CH), 120.93 (CH), 130.32 (CH), 133.17 (2C), 138.33 (C), 146.58 (C), 147.71 (C), 155.47 (C), 164.23 (2C), 166.23 (C). HRMS (ESI) (*m*/*z*): [M + H]^+^ calcd. for C_23_H_23_N_3_O_5_S: 454.1431, found: 454.1427.

*N-(1,3-Benzodioxol-5-ylmethyl)-2-[(5-phenyl-1,3,4-oxadiazol-2-yl)thio]acetamide* (**9**). White solid (72%). M.p.: 136 °C. IR ν_max_ (cm^−1^): 3309.85 (N–H stretching), 3072.60 (Aromatic C–H stretching), 2914.44 (Aliphatic C–H stretching), 1670.35 (C=O stretching), 1558.48, 1487.12, 1467.83 (N–H bending, C=N and C=C stretching), 1440.83, 1390.68 (C–H bending), 1315.45, 1251.80, 1230.58, 1197.79, 1178.51, 1097.50, 1068.56, 1039.63 (C–N, C–O stretching and aromatic C–H in plane bending), 939.33, 921.97, 889.18, 869.90, 806.25, 773.46, 702.09, 686.66, 657.73 (aromatic C–H out of plane bending and C–S stretching). ^1^H-NMR (400 MHz, DMSO-*d*_6_) δ (ppm): 4.17 (s, 2H, S-CH_2_), 4.23 (d, *J* = 5.6 Hz, 2H, Ar-CH_2_), 5.95 (s, 2H, O-CH_2_-O), 6.72–6.82 (m, 3H, aromatic), 7.56–7.65 (m, 3H, aromatic), 7.94–7.96 (m, 2H, aromatic), 8.79 (t, *J* = 5.6 Hz, *J* = 11.2 Hz, 1H, N-H). ^13^C-NMR (75 MHz, DMSO-*d*_6_): 36.13 (CH_2_), 42.91 (CH_2_), 101.27 (CH_2_), 108.41 (2CH), 120.95 (CH), 123.46 (C), 126.81 (2CH), 129.85 (2CH), 132.44 (CH), 133.17 (C), 146.58 (C), 147.71 (C), 163.85 (C), 165.55 (C), 166.49 (C). HRMS (ESI) (*m*/*z*): [M + H]^+^ calcd. for C_18_H_15_N_3_O_4_S: 370.0856, found: 370.0844.

### 3.2. Cytotoxicity Test

NIH/3T3 Mouse embryonic fibroblast (ATCC^®^ CRL-1658™, American Type Culture Collection (ATCC), Manassas, VA, USA), A549 human lung adenocarcinoma (ATCC^®^ CCL-185™, American Type Culture Collection (ATCC), Manassas, VA, USA) and C6 rat glioma (ATCC^®^ CCL-107™, American Type Culture Collection (ATCC), Manassas, VA, USA) cell lines were used for cytotoxicity tests. NIH/3T3 cell line was used to investigate the selectivity of the compounds, whereas other cell lines were used to determine anticancer activity. All cell lines were incubated according to the instructions of the supplier at 37 °C in a humidified atmosphere of 95% air and 5% CO_2_. NIH/3T3, A549 and C6 cells were seeded at 1 × 10^4^ cells into each well of 96-well plate. After 24 h of incubating period, the culture mediums were removed and compound was added to culture medium at 500–3.9 μg/mL doses with a dilution factor of 2 (3.9, 7.8, 15.6, 31.2, 62.5, 125, 250, 500 μg/mL). After 24 h of incubation, XTT (2,3-bis(2-methoxy-4-nitro-5-sulfophenyl)-2*H*-tetrazolium-5-carboxanilide) assay was performed using the In Cytotox-XTT 1 Parameter Cytotoxicity Kit (Xenometrix, Allschwil, Switzerland) as previously described [61]. Inhibition% was calculated for each concentration of the compounds according to the formula below obtained from the instructions of the manufacturer and IC_50_ values were estimated by plotting a dose response curve of the inhibition% versus test compound concentrations. All experiments were done in quadruplicates at three different time points, and the results were given as Mean ± standard deviation. Cisplatin was used as a positive control. The stock solutions of the compounds were prepared in dimethyl sulfoxide (DMSO) and further dilutions were made with fresh culture medium. The final DMSO concentration was under 0.1%.

$$\mathrm{Inhibition}\%=100-\left(\;\frac{\mathrm{corrected}\;\mathrm{mean}\;\mathrm{OD}\;\mathrm{sample}}{\mathrm{corrected}\;\mathrm{mean}\;\mathrm{OD}\;\mathrm{solvent}\;\mathrm{controls}}\;\times 100\right)$$

SI was also calculated to compare the selectivity of the compounds according to a previous study [62] as follows:

$$\mathrm{SI}=\frac{{\mathrm{IC}}_{50}\mathrm{value}\;\mathrm{for}\;\mathrm{NIH}/3T3\;\mathrm{cell}\;\mathrm{line}}{{\mathrm{IC}}_{50}\mathrm{value}\;\mathrm{for}\;A549\;\mathrm{or}\;C6\;\mathrm{cell}\;\mathrm{line}}\;\times 100$$

IC_50_ value greater than any value (>value; as seen in Table 1) was taken as the value itself to calculate SI value.

### 3.3. Matrix Metalloproteinase (MMP) Inhibition Assays

MMP-1, -2, -8, -9, -13 colorimetric kits were purchased from Enzo Life Sciences Inc. (Farmingdale, New York, NY, USA). The MMP Colorimetric Drug Discovery Kits are complete assay system designed to screen MMP inhibitors using a thiopeptide as a chromogenic substrate (Ac-PLG-[2-mercapto-4-methyl-pentanoyl]-LG-OC_2_H_5_). The MMP cleavage site peptide bond is replaced by a thioester bond in the thiopeptide. Hydrolysis of this bond by a MMP produces a sulfhydryl group, which reacts with DTNB [5,5′-dithiobis(2-nitrobenzoic acid), Ellman’s reagent] to form 2-nitro-5-thiobenzoic acid, which can be detected by its absorbance at 412 nm. The assays were conducted in triplicate. The UV absorbance was read at 412 nm using a microplate reader (BioTek, PowerWave, Gen5 software, Winooski, VT, USA) at room temperature. NNGH was used as a control inhibitor. Data was expressed as Mean ± SD [63].

The inhibitor% remaining activity of MMPs was calculated using the following equation:

Inhibitor% activity remaining = (V inhibitor/V control) × 100

The inhibition (percent) of MMPs was calculated using the following equation:

I (%) = 100 − Inhibitor% activity remaining

### 3.4. Docking Studies

Compounds **8** and **9** were docked to the active site of 5I12. Ligands were set to the physiological pH (pH = 7.4) at the protonation step and crystal structure of MMP-9 was retrieved from Protein Data Bank server (PDB code: 5I12). The structures of compounds **8** and **9** were submitted in protein preparation module of Schrodinger’s Maestro molecular modeling package. In molecular docking simulations: Glide/XP docking protocols were applied for the prediction of topologies of compounds **8** and **9** at the active site of target structure [64,65]. The formation of active site in the enzyme was determined from previous studies [46,57].

## 4. Conclusions

In the present study, we described the synthesis of new oxadiazole, thiadiazole, and triazole derivatives and focused on their antiproliferative effects on A549 human lung adenocarcinoma, C6 rat brain glioma, and NIH/3T3 mouse embryonic fibroblast cell lines. Among these compounds, compounds **1**, **8** and **9** showed notable cytotoxic activity against C6 cell line, whereas compound **8** was found to be the most promising anticancer agent against A549 cell line. The mechanistic studies were performed to investigate the inhibitory effects of these agents on gelatinases (MMP-2, MMP-9) and collagenases (MMP-1, MMP-8, MMP-13). Compounds **8** and **9** were found as the most effective MMP-9 inhibitors in this series. Moreover, these agents did not interfere with MMP-8, the protective MMP enzyme in cancer. Docking studies were carried out for compound **8** and **9** on MMP-9 to enlighten the exact positions and interactions of these compounds on the active site of the MMP-9 enzyme. According to docking studies, compounds **8** and **9** showed good binding affinity to the active site of the MMP-9 enzyme.

## Figures and Tables

**Table 1:** Cytotoxic effects of compounds **1**–**9** and cisplatin on A549, C6, and NIH/3T3 cells.

Compound	IC_50_ Values for Cell Lines (mM)			SI Values ^1^	
	NIH/3T3	A549	C6	A549	C6
**1**	0.308 ± 0.037	>1.234	0.128 ± 0.027	24.959	240.625
**2**	1.413 ± 0.094	>1.432	>1.432	98.673	98.673
**3**	1.369 ± 0.074	1.369 ± 0.058	0.507 ± 0.049	100	270.018
**4**	1.285 ± 0.090	>1.285	>1.285	100	100
**5**	>1.066	>1.066	>1.066	100	100
**6**	>1.211	>1.211	>1.211	100	100
**7**	0.100 ± 0.011	>1.166	>1.166	8.576	8.576
**8**	>1.104	0.125 ± 0.020	0.137 ± 0.015	883.200	805.839
**9**	0.888 ± 0.029	0.349 ± 0.046	0.157 ± 0.016	254.441	565.605
**Cisplatin**	>1.166	0.140 ± 0.030	0.103 ± 0.026	1190	1617.476

^1^ SI values were calculated from the equation as follows: $\mathrm{SI}=\frac{{\mathrm{IC}}_{50}\mathrm{value}\;\mathrm{for}\;\mathrm{NIH}/3T3\;\mathrm{cell}\;\mathrm{line}}{{\mathrm{IC}}_{50}\mathrm{value}\;\mathrm{for}\;A549\;\mathrm{or}\;C6\;\mathrm{cell}\;\mathrm{line}}\;\times 100$.

**Table 2:** MMPs Inhibition Percentage (%) of compounds **1**, **8** and **9**.

Compound (1.3 μM)	Inhibition % (Mean ± SD)				
	MMP-1	MMP-2	MMP-8	MMP-9	MMP-13
**1**	----	----	15 ± 1.4	24.78 ± 0.86	----
**8**	----	----	----	25.75 ± 2.16	----
**9**	----	----	----	30.46 ± 3.27	----
**NNGH**	87.79 ± 3.49	100	100	88.44 ± 3.35	93.74 ± 3.06
